# Depression and Anxiety Outcomes in a Technology-Enabled Psychotherapy Practice: Retrospective Cohort Study

**DOI:** 10.2196/76264

**Published:** 2025-12-02

**Authors:** Nicholas R Forand, Jasmine Nettiksimmons, Margaret Anton, Raven Truxson, Karl Vanderwood, Brandn Green

**Affiliations:** 1 Two Chairs San Francisco, CA United States; 2 JG Research & Evaluation Bozeman, MT United States

**Keywords:** depression, anxiety, mental health, mental health care, measurement-based care, psychotherapy

## Abstract

**Background:**

Mental health conditions account for significant distress, burden, and societal costs. Despite efforts to implement evidence-based practices (EBPs), access to high-quality mental health treatment in general practice remains limited and clinical outcomes suboptimal. Measurement-based care (MBC) is a transtheoretical and transdiagnostic strategy that has the potential, when implemented effectively, to improve the quality of care. Digital tools can also support clinicians by alleviating administrative tasks and providing in-the-moment performance data and clinical decision support.

**Objective:**

This study aimed to examine patient retention in care and depression and anxiety outcomes in a technology-enabled psychotherapy practice where clinicians were supported by a suite of innovations, including an MBC platform and clinical decision support tools.

**Methods:**

This retrospective cohort study examined 2984 adults who initiated mental health treatment with Two Chairs, a hybrid technology–enabled behavioral health provider, from January 1 to June 30, 2024. Rates of reliable change, recovery, remission, deterioration, and the magnitude and trajectory of symptom change in depression and anxiety symptoms were assessed using the Patient Health Questionnaire-9 (PHQ-9) and General Anxiety Disorder-7 (GAD-7).

**Results:**

The participants demonstrated high rates of retention in care (3183/3572, 89.1%) and MBC survey completion (3440/3572, 96.3%). From baseline to the 12th session, patients showed significant symptom improvements in depression and anxiety, achieving high rates of reliable improvement (65.8%, 95% CI 64.0%-67.5%) and recovery (53.2%, 95% CI 51.4%-55.0%), with 71.3% (95% CI 69.7%-72.9%) of patients achieving either outcome. Outcomes continued to improve up to the point of completion of the treatment episode (79.2%, 95% CI 77.5%-80.9%, of patients achieving improvement or recovery). Pre- to posttreatment effect sizes were large (all Cohen d>0.9).

**Conclusions:**

The results of this study show that technology-enabled MBC and clinical decision support systems (CDSSs) may support high-quality patient outcomes in mental health.

## Introduction

### Background

Mental health conditions are a significant public health concern, contributing substantially to the global burden of disease [1]. The increased prevalence of mental health disorders has a clear relationship with disability, reduced quality of life, and associated increases in avoidable economic costs, including costs incurred by payers while managing the care of individuals with these conditions [2,3]. The economic burden of mental illness is high, affecting both direct health care expenditures and indirect costs connected to loss of productivity in the workforce and increased demand for social supports. An analysis of the change in the economic burden of major depressive disorder between 2010 and 2018 describes a worldwide increase from US $236.6 billion to US $326.2 billion [2]. These broad and significant personal, social, and economic implications underscore the urgency of addressing mental health challenges [4].

### Access to Quality Care

Despite a substantial increase in the percentage of US adults receiving mental health services over the past 25 years, access to effective, high-quality mental health care remains problematic [4]. Studies examining early termination of psychotherapy treatment suggest that a significant portion of patients drop out of therapy prematurely [5,6]. One study found that 73.5% of patients in a health system setting dropped out of care prior to their fourth session, suggesting that the majority of patients received an inadequate dose of treatment [6]. High levels of treatment dropout have been attributed to low therapeutic alliance and dissatisfaction with the quality of care [7].

A systematic review of usual care practices highlighted that many mental health interventions also lack a structured, evidence-based approach, leading to inconsistent treatment delivery and variable patient outcomes [8,9]. Outcomes in regular care practices reflect these problems, with rates of improvement being up to 20%-30% lower in these settings than in clinical trials of structured, evidence-based interventions [10-12].

Although there has been a growing recognition of mental illness as a critical health issue, the quality of care provided to individuals with mental disorders remains suboptimal due to limited quality standards, weak therapeutic alliance, and underuse of evidence-based approaches, all of which further contribute to the societal burden of mental illness.

### Barriers to Effectively Implementing Evidence-Based Practices

Evidence-based practices (EBPs) are known to improve patient outcomes; however, the implementation of these practices in mental health care can be hindered by cost, complexity, and challenges in sustaining the new clinical model [13,14]. Although standard models of scaling EBPs can be effective, they often require significant financial investment, clinician support, and logistical coordination, limiting their long-term viability [13]. Institutions interested in high-quality care require effective EBPs, as well as efficient and cost-effective means to support clinicians in delivering these practices [15].

### Potential of Measurement-Based Care

Measurement-based care (MBC), sometimes referred to as routine outcome monitoring or feedback-informed care, is an EBP that includes the collection, sharing, and use of patient-reported data to collaboratively guide clinical decision-making [16,17]. When implemented effectively, MBC has been demonstrated to improve clinical outcomes [17]. Unlike other evidence-based mental health interventions that are often designed to target a specific clinical diagnosis or concerns in targeted populations, MBC is both transtheoretical and transdiagnostic, suggesting that it is an efficient method for improving outcomes across diverse groups of patients and clinicians. Research indicates that MBC enhances therapy outcomes by enabling clinicians to identify and address care delivery and alliance challenges in real time, leading to reduced dropout [18], improved therapeutic alliance, faster symptom resolution, and a more personalized, effective approach than standard care [17].

Despite these advantages, the implementation of MBC also faces challenges. At the clinician level, the time and administrative burden associated with selecting, delivering, scoring, and tracking scores over time may inhibit uptake [19]. Without support, clinicians may also struggle with generating insights from MBC data or tracking and identifying trends. At the organizational level, leadership alignment, funding, the implementation of new technology, training, and supervision are cited as limitations to adoption [19]. Barriers to the implementation of MBC may be reflected in the rates of clinicians who use these tools. Less than 20% of clinicians indicate using MBC in their practice [20], and even when measures are prescribed by the clinical service, rates of survey completion can be as low as 15% [21].

### Technology as a Solution to MBC Barriers

Clinical decision support systems (CDSSs) and other clinical dashboard applications offer technology-enabled solutions to address many of the aforementioned challenges, including the implementation of MBC [16,17]. A CDSS is a form of technology supporting care that can be integrated into health care settings. It can improve patient outcomes and relieve the clinician burden by providing clinicians with evidence-based recommendations at the point of care [20]. CDSSs analyze patient data and suggest appropriate interventions and follow-up, thereby enhancing clinical efficiency and reducing clinical errors [18]. By integrating these systems, clinicians are able to make informed decisions that align with the latest clinical guidelines, leading to improved patient outcomes [20].

Technology can also alleviate administrative tasks, allowing clinicians to focus more on patient care [19]. Additionally, digital tools can facilitate real-time performance feedback, enhancing visibility and accountability across all levels of care [21]. Clinicians can monitor patient progress, symptom changes, and treatment responses [22]. In some instances, patients can also track their own progress, empowering them in their own care [22]. Systematic collection and feedback of data can also be leveraged for quality improvement efforts, such as monitoring clinician or practice performance and identifying areas for remediation or professional development [22]. Across this broad set of supports, technological innovations in care delivery, patient tracking, and analysis have demonstrated the capacity to increase sustainability of services through enhanced quality and patient engagement, while also decreasing the administrative burden associated with documenting the provision of care. MBC paired with CDSS support may provide a means to improve the efficiency and sustainability of EBP.

Despite the promise of this approach, technology-enabled psychotherapy is not without potential limitations. Design and usability challenges are common in the technology space. When a digital CDSS tool is not perceived by users to be usable, valid, and useful in clinical practice, it can limit adoption or engagement and may even add an additional administrative burden to the workforce [23-25]. CDSSs are more likely to be usable and acceptable if designed with clinician input or “user-centered design” processes. Thoughtfully co-designed CDSS tools can help more effectively realize the benefits of technology-enabled clinical practice.

### Aims of This Study

Technology-supported psychotherapy practice, featuring MBC supported by a suite of CDSS tools, may improve the scalability of high-quality care in general psychotherapy practice. Although the literature provides many examples of how technology may support the practice of MBC, many of these studies are confined to randomized controlled trials (RCTs) or other limited implementations or focus primarily on the effectiveness and practice of MBC [26]. This study aimed to describe a broad suite of CDSSs that have been designed and implemented to support clinicians in general practice in the delivery of MBC and other aspects of care. To evaluate the general outcomes and clinical processes associated with practice using this suite of tools, the study reported on the following markers of clinical quality: (1) patient retention in care (ie, reduced dropout); (2) patient and clinician adherence to MBC practices; and (3) clinical outcomes, including depression and anxiety symptom reduction for individuals who enter care with clinically elevated symptoms. The study used a retrospective cohort design and included patients who engaged in weekly outpatient psychotherapy, either virtually or in person. The study’s findings will contribute to the limited literature on the real-world use of technology-supported MBC practice at scale.

## Methods

### Ethical Considerations

The need for formal ethical approval was waived by the Sterling Institutional Review Board (IRB), Atlanta, Georgia. This research was deemed exempt under Department of Health and Human Services (DHHS) Category 4 as retrospective research on an existing and de-identified dataset. The IRB protocol number is 12670-NForand.

### Study Design and Study Setting

This was a retrospective cohort analysis focused on adult patients with clinically elevated symptoms of depression or anxiety who enrolled in mental health treatment at Two Chairs in the first half of 2024. Two Chairs is a technology-enabled behavioral health company founded in 2017 that provides psychotherapy to self-paying individuals, commercially insured (health maintenance organizations [HMOs], preferred provider organizations [PPOs]) individuals, and publicly insured (Medicare, Medicaid) individuals. During the data collection period, services were available in California, Washington, and Florida.

### Participants

The following details describe general facts about the practice setting. Adult patients (≥18 years old) are enrolled in behavioral health services at Two Chairs and receive outpatient psychotherapy for anxiety, depression, and related conditions. At intake, the patients complete a digital intake assessment and attend an initial clinical interview, after which they are assigned a preliminary diagnosis and subsequently matched with an individual clinician. During onboarding, 98% of all patients are screened for depression and anxiety using the Patient Health Questionnaire-9 (PHQ-9) [27] and General Anxiety Disorder-7 (GAD-7) [28], and 48% and 45% of patients meet the clinical cutoff of ≥10 on these measures, respectively. Of those with a positive screen on either measure, 84% start care within 30 days. Of patients who receive a diagnosis of a depressive disorder, 99% have at least one follow-up PHQ-9 assessment, and of those who receive a diagnosis of anxiety disorder, 99% have at least one follow-up GAD-7 assessment, indicating a high level of follow-up assessment.

The study required patients to have a first treatment session between January 1 and June 30, 2024. Patients were eligible for the study if they had clinical levels of depression or anxiety at their baseline measurement, as evidenced by a PHQ-9 or a GAD-7 score of ≥10. Using symptom cutoffs is common in similar studies of general practice [29] and ensures that patients are likely to have clinically elevated levels of distress and are therefore in need of treatment. Patients reporting active suicidal ideation or otherwise deemed inappropriate for outpatient psychotherapy at intake were referred to health plan or community resources and not included in this study. The cutoff for data included in this study was February 28, 2025 regardless of whether a patient had completed their care by that date. A total of 3572 patients met the initial eligibility criteria.

Patients were excluded from the primary analytic cohort if they terminated from care early (defined as fewer than four sessions), missed a symptom assessment at session 1, or lacked any follow-up symptom assessments between sessions 4 and 12. The cutoff for inclusion of at least four attended sessions was used to ensure the sample had an adequate dose of therapy. The four-session cutoff has been used previously in other large studies of naturalistic care [30,31]. To ensure a comprehensive report of the outcomes for the full cohort, the outcomes of individuals who dropped out prior to four sessions were included as a separate analysis. After these criteria were applied, the primary analytic cohort comprised 2984 (83.5%) patients. Within the primary cohort, inclusion criteria for PHQ-9 outcomes (baseline PHQ-9 score≥10) were met by 2465 (82.6%) patients, and inclusion criteria for GAD-7 outcomes (baseline GAD-7 score≥10) were met by 2204 (73.9%) patients. A secondary analysis focused on the subset of patients from the primary cohort who had completed their course of care during this time frame (n=2237, 75%; PHQ-9 n=1812, 81%; GAD-7 n=1689, 75.5%).

### The Two Chairs Teletherapy Intervention

Patients enrolled at Two Chairs receive weekly 45-55-minute teletherapy or in-person mental health services delivered by licensed clinicians. Approximately 99% of the sessions are conducted via teletherapy. Clinicians use a range of treatment modalities to address patients’ needs, including but not limited to cognitive behavioral therapy, acceptance and commitment therapy, time-limited dynamic psychotherapy, and somatic therapy. As part of their practice, clinicians are required to use MBC to evaluate patients’ symptom presentation, make care decisions, and track progress in care. Prior to each session, patients complete measures of depression (PHQ-9) and anxiety (GAD-7) symptoms, mental health quality of life (MHQoL), and therapeutic alliance. All symptom data are derived from the regular MBC assessments. For these analyses, only the PHQ-9 and GAD-7 results are reported.

**Figure 1:**
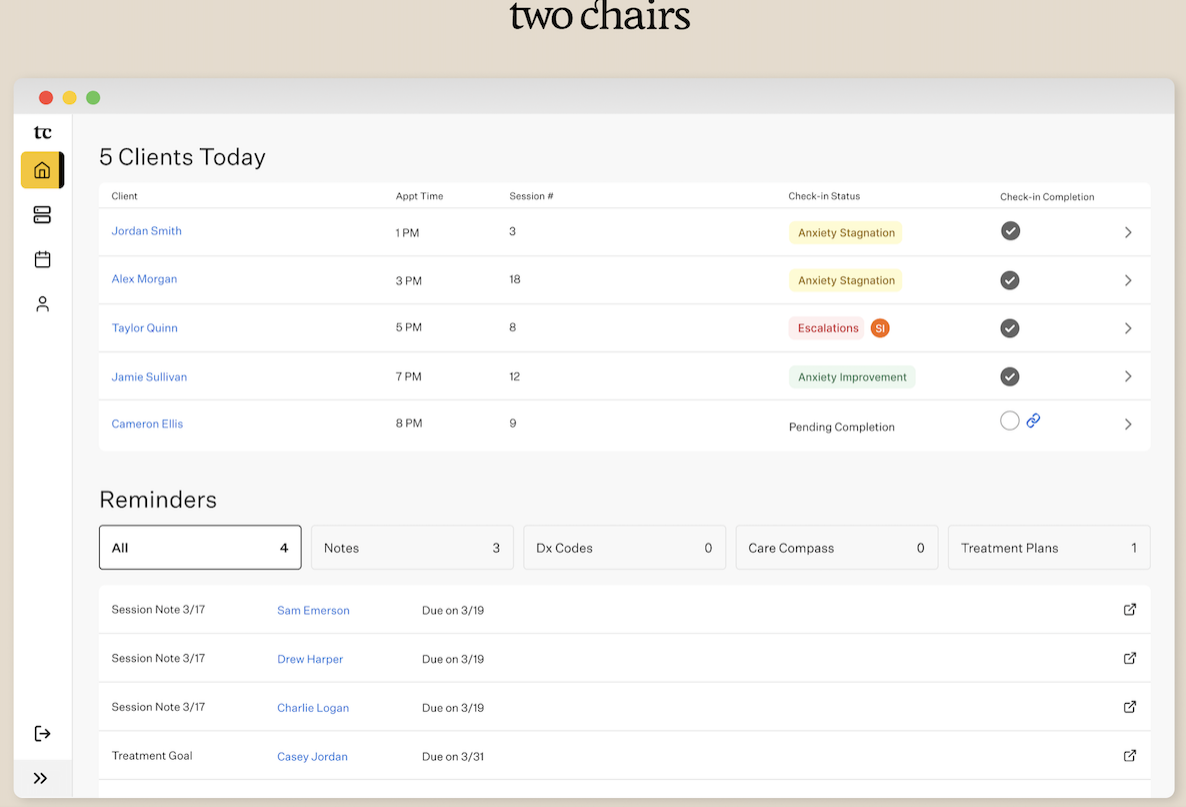
Image of the Two Chairs clinical dashboard for tracking patients.

**Figure 2:**
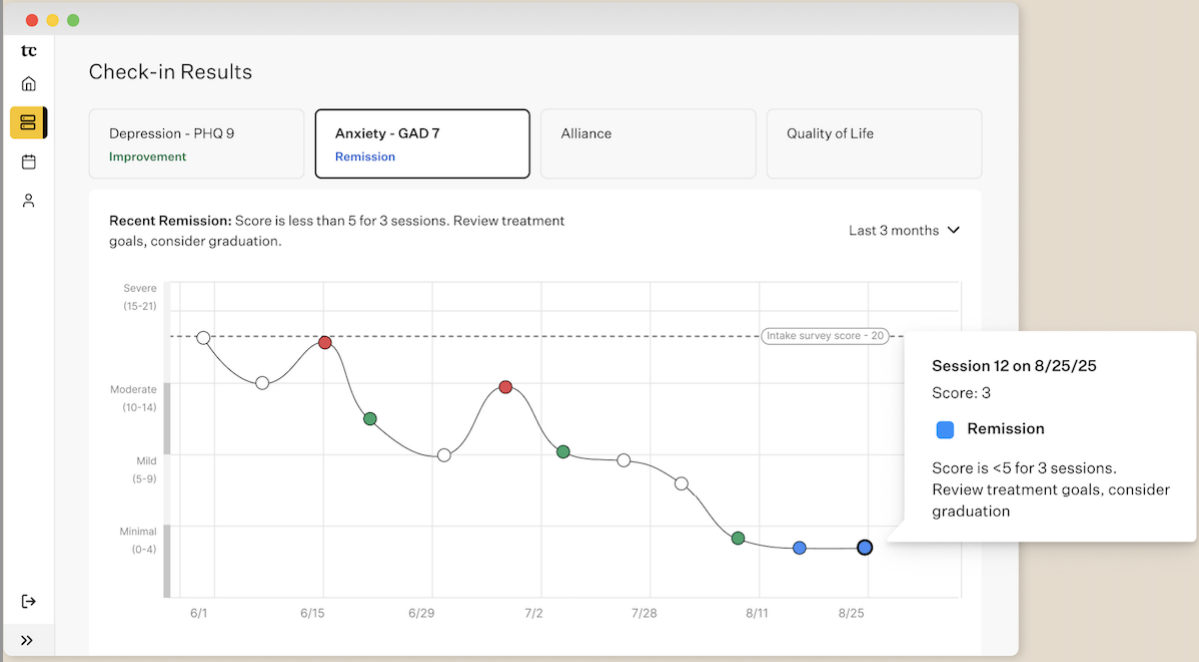
View of GAD-7 scores on the Two Chairs patient page. GAD-7: General Anxiety Disorder-7.

**Figure 3:**
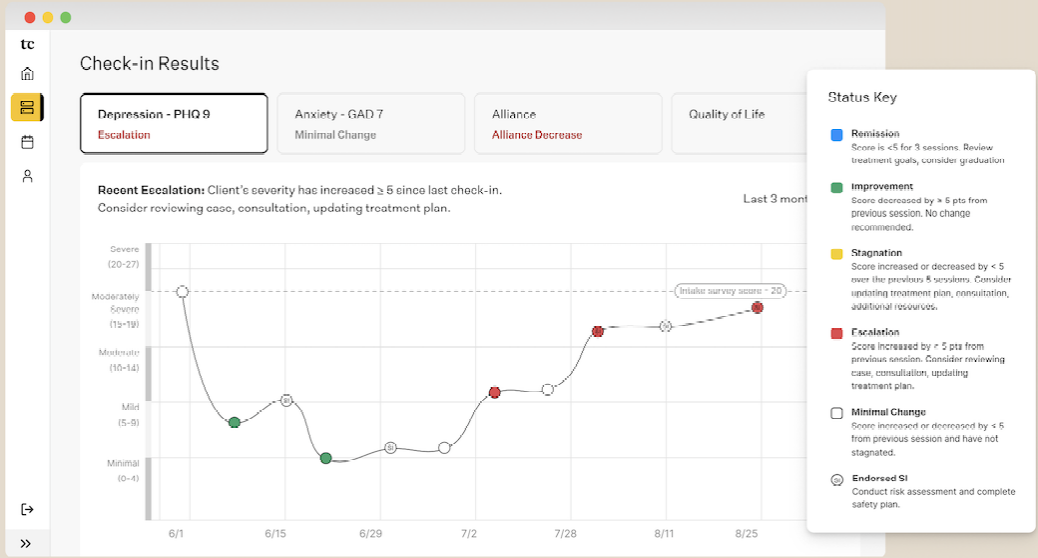
View of PHQ-9 scores on the Two Chairs clinician patient page. PHQ-9: Patient Health Questionnaire-9.

**Figure 4:**
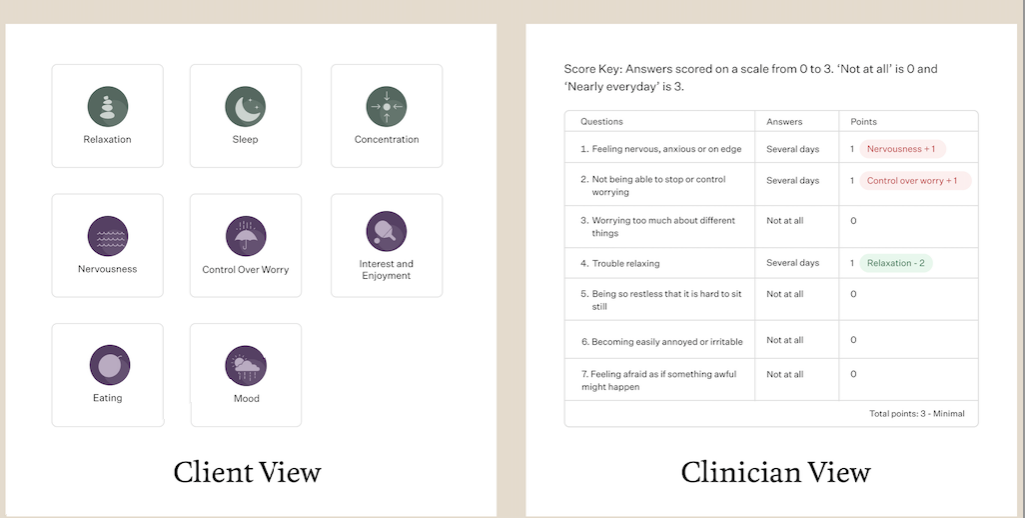
View of patient-facing MBC feedback and Two Chairs clinician-facing tabular view. MBC: measurement-based care.

Following standard user-centered design principles, all CDSS tools provided by Two Chairs have been co-designed with user input and tested and revised before launching to the full clinical population. Satisfaction with clinical tools is assessed quarterly and these data are used to drive software and tooling improvements. The product and clinical teams engage in regular review and revision of these tools to ensure they are meeting user needs and adhering to clinical best practices. The technological supports offered by Two Chairs include (1) automated electronic MBC questionnaire delivery to patients; (2) automated scoring and interpretation of patient-reported symptoms over time; (3) clinician alerting for potential safety and risk concerns, such as endorsement of suicidal ideation; and (4) clinical decision support features, including indications of clinically meaningful symptom changes or changes in alliance, standardized recommendations for clinician actions, alerts related to patient-endorsed suicidal ideation, and holistic summaries of change across all measures (see Figures 1-4). Patients also provide a summary of their symptoms, along with highlights of meaningful symptom change, to allow them to reflect on their care progress and feel empowered to engage in care decisions. Clinicians have access to patient goals, diagnoses, and number of sessions on each patients’ individual page within the platform. All clinical data are graphically displayed prominently on the home page for each patient on Two Chairs’ software platform.

Upon onboarding to Two Chairs, all clinicians receive a full digital training series on the use of MBC in practice, and orientation to Two Chairs tools. Clinicians are trained to integrate patient reported MBC data into care and engage in collaborative decision-making with patients around their treatment progress. The full training package is described in a separate paper [32]. Adherence to MBC practices are tracked by the software system and are part of clinician performance reviews.

### Measurement and Variables

#### Patient Demographics

As part of the onboarding process at Two Chairs, patients provided basic demographic information (eg, age, race/ethnicity, gender, employment status). Data were collected as part of routine care, and patients were not required to complete all questions. As a result, data completeness varied.

#### Patient Retention

Patient retention was evaluated as the proportion of patients who met the initial study inclusion criteria and were retained until session 4, qualifying for inclusion in the primary analytic cohort.

#### MBC Completion

MBC completion was assessed as the proportion of observed sessions (up to 12) in which MBC assessments were completed across the entire included population. To assess how well individual clinicians adhered to MBC procedures, we also determined the average completion rates of MBC surveys for patients included within each clinician’s panel. The result was a clinician-level assessment of MBC completion for all of their included patients.

#### Measures

Patients completed the PHQ-9 and GAD-7 at each session throughout care. The PHQ-9 is a 9-item self-report measure of depressive symptoms. Items are rated on a scale ranging from 0 (not at all) to 3 (nearly every day). Total scores range from 0 to 27, and cutoff scores for mild, moderate, moderately severe, and severe depressive symptoms are 5, 10, 15, and 20, respectively. A reliable change on the PHQ-9 is a change of ≥5 in the PHQ-9 score [33]. The GAD-7 is a 7-item self-report measure of anxiety. Items are rated on a scale ranging from 0 (not at all) to 3 (nearly every day). Total scores range from 0 to 21, and the cutoff scores for mild, moderate, and severe anxiety symptoms are 5, 10, and 15, respectively. A reliable change on the GAD-7 is a change of ≥4 in the GAD-7 score [34].

### Outcomes

#### Clinical Outcomes

The number of sessions delivered at Two Chairs varies based on patient presentation; therefore, we used the 12th session of care as a common end point in order to standardize outcomes measurement for the primary analyses. The use of the 12th session as a standard end point in open-ended therapy models is consistent with other studies [11,35]. Baseline clinical scores were derived from the first therapy session. Clinical outcomes were described at two time points: the clinical “check-in” session (defined as session 12 or the last available session between sessions 4 and 11 with a symptom assessment) and termination (defined as the last available symptom measurement for patients who completed their course of care). The latter sample included patients who completed their course of care at any session prior to February 28, 2025. Outcomes were also presented for those patients who dropped out prior to the fourth session, provided those patients met all other inclusion criteria and had at least two observations (n=213).

This study focused on characterizing depression and anxiety symptom improvement using both joint outcomes for the PHQ-9 and GAD-7 and independent outcomes for the PHQ-9 and GAD-7. The goal of the joint measures was to holistically evaluate change in the commonly comorbid syndromes of depression and anxiety [36], similar to methods used in other settings where these measures are used concurrently [33]. Patients were included in the summaries of joint outcomes if either the PHQ-9 or the GAD-7 baseline score was ≥10. For assessment-specific outcomes (PHQ-9 or GAD-7), inclusion required a baseline score of ≥10 on the given assessment.

#### Independent Outcomes

Regarding assessment-specific outcomes, *reliable improvement* was defined as an improvement of 5 points on the PHQ-9 or 4 points on GAD-7; *reliable deterioration* was defined as a worsening of 5 points on the PHQ-9 or 4 points on GAD-7; *recovery* was defined as a follow-up score of <10; *remission* was defined as a follow-up score of <5; and *response* was defined as a 50% or greater improvement in the score from baseline.

#### Joint Outcomes

For joint measures, reliable improvement was defined as reliable improvement on either the PHQ-9 or GAD-7, without reliable deterioration on the other measure. Joint reliable deterioration was defined as reliable deterioration on at least one measure, regardless of a change in the other measure. Recovery was defined as a PHQ-9 and GAD-7 score of <10 at follow-up. Clinically meaningful improvement was defined as meeting either joint recovery or joint reliable improvement criteria. This holistic measure of change across both depression and anxiety encompasses both magnitude of change and symptom severity at the time of measurement.

### Statistical Analysis

The study population was characterized using means (SDs) and frequencies with statistical tests to evaluate differences between the primary cohort and the population that was excluded from analysis (*t* tests, chi-squared tests, Fisher exact tests for cell counts<20).

PHQ-9 and GAD-7 scores were measured at baseline, the check-in session, and termination with means (SDs). Change from baseline on the PHQ-9 and GAD-7 was described by the mean difference, 95% CIs, and Cohen d. Summaries of dichotomous outcomes (reliable improvement, recovery, meaningful improvement, remission, and response) were described with frequencies and 95% CIs.

Longitudinal analysis of the PHQ-9 and GAD-7 was performed using linear mixed effects models with intercepts for clinicians and random intercepts and slopes (session numbers) for patients. Fixed effects included age, gender, the session number, and a quadratic term for the session number (see Multimedia Appendix 1). Average effects across sessions were estimated with the most commonly occurring level of age and gender. There were no missing values for age. Missing entries for gender were addressed with an explicit parameter for gender missingness. Missing PHQ-9 or GAD-7 data at the session level were handled within the modeling framework using maximum likelihood estimation.

All analyses were performed using R version 4.4.2 (2024-10-31; R Foundation for Statistical Computing). All data used in the study were extracted from the Two Chairs electronic health record system. Study authors used the STROBE (Strengthening the Reporting of Observational Studies in Epidemiology) checklist to ensure complete methodological and scientific content was provided in the manuscript for an observational study. The checklist is included in Multimedia Appendix 2.

## Results

### Patient Characteristics

**Figure 5:**
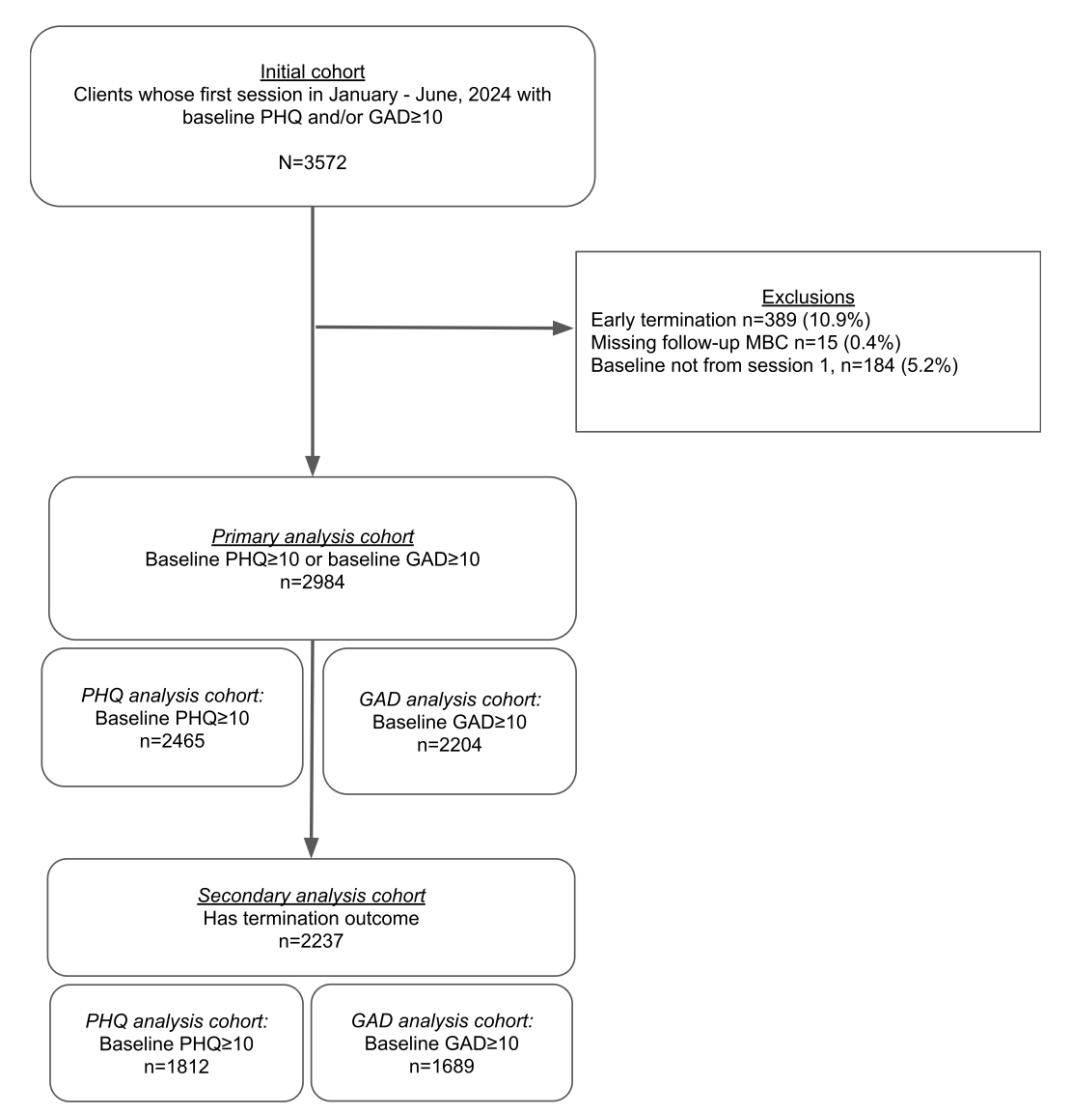
CONSORT (Consolidated Standards of Reporting Trials) diagram on sample size and study participant inclusion. GAD: general anxiety disorder; MBC: measurement-based care; PHQ: Patient Health Questionnaire.

Figure 5 shows the CONSORT (Consolidated Standards of Reporting Trials) guidelines–based diagram on study participant inclusion. Of the 3572 patients who met the initial criteria for inclusion, 89.9% (n=3183) were retained for the fourth session and were thus eligible for the primary analytic cohort. A small number of patients were excluded due to missing follow-up MBC scores or because their session 1 baseline was unavailable, leaving 2984 patients (83.5% of the population meeting the original inclusion criteria; Figure 5).

Patient demographics can be found in Table 1. Data for the clinical check-in session were most often collected from session 12 (n=2311, 71.4%) and the average check-in session number of 10.8. A total of 2237 (75%) patients from the primary cohort terminated care during the observation window and therefore had termination outcomes. Of the patients who completed their care, 38.1% (n=852) did so by session 12 and 61.9% (n=1385) did so after session 12. The median termination session was 16 (average 16.9).

**Table 1:** Demographics and baseline characteristics of patients included and excluded in the primary cohort.

Characteristics		Patients included (n=2984)	Patients excluded (n=588)	*P* value
**Age (years), n (%)**				
	18-29	774 (25.9)	134 (22.8)	.05
	30-49	1549 (51.9)	295 (50.2)	.05
	50-69	545 (18.3)	127 (21.6)	.05
	≥70	116 (3.9)	32 (5.4)	.05
**Gender, n (%)**				
	Woman	1793 (60.1)	361 (61.4)	.006
	Man	695 (23.3)	123 (20.9)	.006
	Unknown	325 (10.9)	85 (14.5)	.006
	Trans/nonbinary/other	171 (5.7)	19 (3.2)	.006
**Race and ethnicity, n (%)**				
	Non-Hispanic White	1297 (43.5)	231 (39.3)	.23
	Hispanic	573 (19.2)	118 (20.1)	.40
	Unknown race/ethnicity	544 (18.2)	141 (24.0)	.001
	Asian	371 (12.4)	59 (10.0)	.20
	Black	273 (9.1)	61 (10.4)	.26
	Other race/ethnicity	250 (8.4)	47 (8.0)	.99
	American Indian/Alaska Native	80 (2.7)	18 (3.1)	.49
	Asian Pacific Islander	74 (1.5)	9 (1.5)	.23
**Primary payer, n (%)**				
	HMO^a^	2316 (77.6)	408 (69.4)	<.001
	Public (Medicare, Medicaid)	478 (16.0)	123 (20.9)	<.001
	Commercial insurance (PPO^b^)	142 (4.8)	38 (6.5)	<.001
	Self-pay	48 (1.6)	19 (3.2)	<.001
**Employment status, n (%)**				
	Full-time employee	1615 (54.1)	293 (49.8)	.01
	Other	774 (25.9)	139 (23.6)	.01
	Unknown	315 (10.6)	84 (14.3)	.01
	Disabled/unemployed	280 (9.4)	72 (12.2)	.01
**Diagnosis category, n (%)**				
	Anxiety and fear-related disorders	990 (33.2)	178 (30.3)	<.001
	Depressive disorders	971 (32.5)	176 (29.9)	<.001
	Trauma- and stressor-related disorders	422 (14.1)	61 (10.4)	<.001
	Adjustment disorders	289 (9.7)	62 (10.5)	<.001
	Other or primary diagnosis unknown	312 (10.5)	111 (18.9)	<.001
**Baseline score, mean (SD)**				
	PHQ-9^c^	13.2 (4.6)	13.3 (5.1)	.70
	GAD-7^d^	12 (4.2)	12.6 (4.3)	.001

^a^HMO: health maintenance organization.

^b^PPO: preferred provider organization.

^c^PHQ-9: Patient Health Questionnaire-9.

^d^GAD-7: General Anxiety Disorder-7.

### MBC Adherence

**Figure 6:**
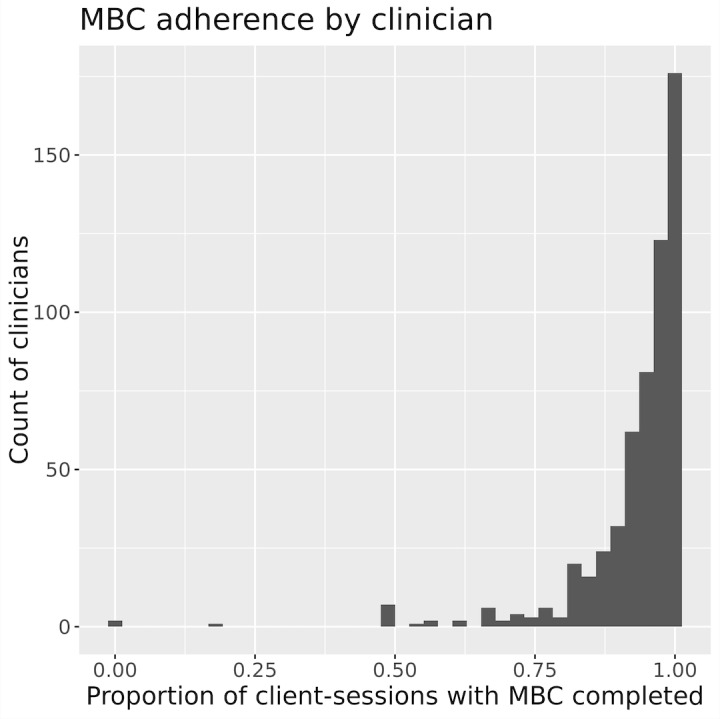
MBC adherence by clinician. MBC: measurement-based care.

Patients in the primary cohort completed the Two Chairs MBC assessments 96.3% (3440/3572) of the time, leading up to the clinical check-in session, which provided near universal weekly data on depression, anxiety, the quality of life, and therapeutic alliance. Among clinicians, the median within-clinician patient adherence to MBC assessments was 96% (IQR 92%-99%). The distribution of within-clinician MBC adherence can be seen in Figure 6.

Within the primary analytic cohort, the population was typically between the ages of 30 and 49 years, with slightly more females, was more commonly employed, and more commonly had an HMO as a payer source. Although 18.2% (n=544) had an unknown race or ethnicity, the sample was notable for the racial diversity represented, with just 43.5% (n=1297) self-identifying as non-Hispanic White (Table 1). The excluded population was more likely to have an unknown/unspecified gender, race, and employment status—all features collected from an optional patient profile—and was more likely to have public insurance and primary diagnoses other than anxiety disorders, depressive disorders, and adjustment disorders. Table 2 shows changes in depressive and anxiety symptoms from baseline to the clinical check-in session (for all individuals included in the primary cohort) and changes from baseline to termination (for individuals included in the secondary cohort). At both the check-in assessment and termination, Two Chairs patients showed improvements with large effect sizes across both cohorts and symptom measures.

**Table 2:** Change in MBC^a^ scores from baseline to check-in (primary cohort) and termination (secondary cohort).

Measure	Baseline	Clinical check-in session					Termination session			
	Mean (SD)	Patients (N=2984), n (%)	Mean (SD)	Change (95% CI)	Cohen d	Patients (N=2237), n (%)		Mean (SD)	Change (95% CI)	Cohen d
PHQ-9^b^	14.5 (3.8)	2465 (82.6)	9.5 (5.5)	–5 (–5.3 to –4.8)	0.92	1812 (81.0)		7.9 (5.7)	–6.6 (–6.9 to –6.3)	1.14
PHQ-9 change (%)	—^c^	—	—	–33.9% (–35.4% to –32.5%)	0.94	—		—	–45.0% (–46.8% to –43.3%)	1.2
GAD-7^d^	13.8 (3.1)	2204 (73.9)	8.7 (5.1)	–5.1 (–5.3 to –4.9)	1.03	1689 (75.5)		7.2 (5.2)	–6.6 (–6.8 to –6.3)	1.25
GAD-7 change (%)	—	—	—	-36.6% (-38.1% to –35.1%)	1.04	—		—	-47.3% (-49.0% to –45.5%)	1.31

^a^MBC: measurement-based care.

^b^PHQ-9: Patient Health Questionnaire-9.

^c^Not applicable.

^d^GAD-7: General Anxiety Disorder-7.

From baseline to the most recent check-in session, patients showed, on average, a 5-point (Cohen d=0.92), 34% (Cohen d=0.94) decrease in PHQ-9 scores and a 5.1-point (Cohen d=1.03), 37% (Cohen d=1.04) decrease in GAD-7 scores. From baseline to termination, patients showed, on average, a 6.6-point (Cohen d=1.14), 45% (Cohen d=1.2) decrease in PHQ-9 scores and a 6.6-point (Cohen d=1.25), 47% (Cohen d=1.31) decrease in GAD-7 scores.

Table 3 shows dichotomous outcomes at the clinical check-in and termination sessions and, where available, across assessment-specific and joint measures of achievement. At the clinical check-in session (typically session 12), 71.3% (95% CI 69.7%-72.9%) of patients achieved clinically meaningful improvement, defined as joint recovery in both anxiety and depression or joint reliable improvement, with improvement in either anxiety or depression, without deterioration on the other. Joint reliable deterioration occurred in 7.9% (95% CI 7.0%-8.9%) of patients. Among patients with a termination outcome available, 79.2% (95% CI 77.5%-80.9%) experienced clinically meaningful improvement, with 6% (95% CI 5%-7%) experiencing reliable deterioration. The majority of patients who met the baseline criteria for PHQ-9 outcomes experienced reliable improvement (53%, 95% CI 51.1%-55.0%) and recovery (55.1%, 95% CI 53.2%-57.1%) on PHQ-9 at the clinical check-in session. These numbers improved to 65.3% (95% CI 63.1%-67.5%) and 66.6% (95% CI 64.4%-68.7%), respectively, among patients with available termination sessions. The majority of patients who met the baseline criteria for GAD-7 outcomes experienced reliable improvement (64.4%, 95% CI 62.4%-66.4%) and recovery (61.5%, 95% CI 59.4%-63.5%) on the GAD-7 measures at the clinical check-in session; both of these measures improved to over 70% at the termination session. Across all measures, outcomes at termination in the secondary cohort were higher than outcomes at clinical check-in for the primary cohort. Outcomes for patients with fewer than four sessions are also included in Table 3 (n=213). Although lower than the outcomes for patients who received an adequate dose of care, 55.4% (95% CI 48.7%-62.1%) of patients who dropped out early also experienced clinically meaningful improvement.

**Table 3:** Patient response, recovery, and remission rates at check-in (primary cohort) and termination (secondary cohort).

Measures		Clinical check-in (primary cohort)	Termination (secondary cohort)	Early terminators (<4 sessions)
		Outcome (95% CI), %	Outcome (95% CI), %	Outcome (95% CI), %
**Joint measures**				
	Clinically meaningful improvement^a^	71.3 (69.7-72.9)	79.2 (77.5-80.9)	55.4 (48.7-62.1)
	Recovery^b^	53.2 (51.4-55.0)	65.2 (63.2-67.2)	35.7 (29.2-42.2)
	Reliable improvement^c^	65.8 (64.0-67.5)	74.8 (73.0-76.6)	47.4 (40.7-54.2)
	Remission^d^	16.4 (15.0-17.7)	27.7 (25.9-29.6)	5.6 (2.5-8.8)
	Reliable deterioration^e^	7.9 (7.0-8.9)	6.0 (5.0-7.0)	10.3 (6.2-14.4)
**PHQ-9^f^ measures**				
	Reliable improvement	53.0 (51.1-55.0)	65.3 (63.1-67.5)	36.6 (29.4-43.8)
	Response^g^	36.4 (34.5-38.3)	51.1 (48.8-53.4)	13.7 (8.6-18.9)
	Recovery	55.1 (53.2-57.1)	66.6 (64.4-68.7)	35.4 (28.3-42.6)
	Remission	19.5 (17.9-21.1)	32.3 (30.2-34.5)	6.9 (3.1-10.6)
	Reliable deterioration	3.5 (2.8-4.2)	3.4 (2.6-4.3)	5.1 (1.8-8.4)
**GAD-7^h^ measures**				
	Reliable improvement	64.4 (62.4-66.4)	73.6 (71.5-75.7)	38.7 (31.1-46.2)
	Response	41.1 (39.0-43.1)	55.8 (53.4-58.1)	14.7 (9.2-20.2)
	Recovery	61.5 (59.4-63.5)	71.9 (69.7-74.0)	37.4 (29.9-44.9)
	Remission	20.8 (19.1-22.5)	34.0 (31.8-36.3)	4.9 (1.6-8.3)
	Reliable deterioration	4.7 (3.8-5.6)	3.9 (3.0-4.8)	3.7 (0.8-6.6)

^a^The patient met the criteria for joint measures for reliable improvement or recovery.

^b^At the clinical end point, both General Anxiety Disorder-7 (GAD-7) and Patient Health Questionnaire-9 (PHQ-9) scores were <10. For individual measures, recovery requires either the PHQ-9 or the GAD-7 score to be <10.

^c^At the clinical end point, the patient showed improvement of at least 5 points on the PHQ-9 or 4 points on GAD-7, without a decline of 5 or 4 points on the other measure, respectively. Reliable improvement requires an improvement of at least 5 points for PHQ-9 measures and at least 4 points for GAD-7 measures.

^d^At the clinical end point, both GAD-7 and PHQ-9 scores were <5. For individual measures, remission requires either the PHQ-9 or the GAD-7 score to be <5.

^e^At the clinical end point, the patient showed an increase of at least 5 points on the PHQ-9 or 4 points on GAD-7. Reliable deterioration requires an increase of at least 5 points for PHQ-9 measures and at least 4 points for GAD-7 measures.

^f^PHQ-9: Patient Health Questionnaire-9. Patients with PHQ-9 scores≥10 at baseline were included.

^g^At the clinical end point, the patient achieved 50% or greater improvement from the baseline score.

^h^GAD-7: General Anxiety Disorder-7. Patients with GAD-7 scores≥10 at baseline were included.

**Figure 7:**
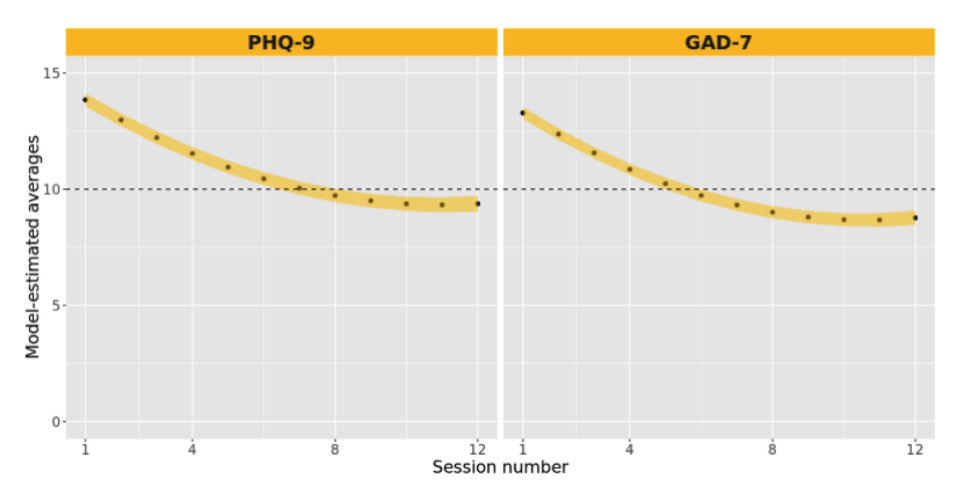
Model-derived estimates for average PHQ-9 and GAD-7 scores over the treatment duration. GAD-7: General Anxiety Disorder-7; PHQ-9: Patient Health Questionnaire-9.

Longitudinal analysis of PHQ-9 and GAD-7 assessments at the session level estimated an approximately 1-point reduction in the assessment score per attended session, which was attenuated over time by approximately 0.05 points per session, accounting for the nonlinear trajectory of change (see Multimedia Appendix 1). Figure 7 shows model-derived average estimates of PHQ-9 and GAD-7 scores across session numbers, along with model parameter-based 95% CIs for each estimated point. The figure demonstrates the swift, early change in symptoms, followed by an attenuated rate of change, with both curves dipping below the clinical threshold (score of 10) near 8 (PHQ-9) and 6 (GAD-7) sessions, respectively.

## Discussion

### Principal Findings

In this study, we examined clinical outcomes among a cohort of 2984 patients who received psychotherapy supported by MBC and a suite of CDSS tools. The study was intended to characterize patient retention and clinical outcomes associated with a large-scale implementation of MBC in a real-world, technology-enabled psychotherapy practice.

The findings indicated that there were clinically meaningful reductions in depression and anxiety symptoms over the course of care among the patients included in the study sample. By the 12th session, 71.3% of patients achieved clinically meaningful improvement in their symptoms, and outcomes improved further when observed at the completion of care (79.2%). On average, improvement in PHQ-9 and GAD-7 scores exceeded the threshold for reliable and clinically significant symptom improvement on these measures [33,34]. We also observed high rates of patient retention in care and over a 95% completion rate for MBC surveys (eg, PHQ-9, GAD-7), indicating high levels of engagement with the care model by clinicians.

Although the study design does not allow us to attribute these outcomes to the suite of CDSS tools or the use of MBC, benchmarks in the literature suggest these outcomes compare favorably to depression and anxiety outcomes achieved in other settings. Meta-analyses of outcomes from RCTs have found depression response rates of 25%-41%, which are lower than the depression response rate of 48% in this study [8,10]. (All Two Chairs outcomes in these comparisons were adjusted to match the inclusion criteria and outcome definitions from the comparator studies. First, we included all individuals who had any follow-up data after baseline in the Two Chairs sample, in contrast to our primary analyses, which included follow-up data only for those who completed at least four sessions. Second, we used a reliable change value of 5 on the GAD-7 for the Blueprint Quality Index comparison to be consistent with its reliable change criteria. Third, we used 12-session outcomes for comparisons to the Blueprint Quality Index comparators, who specified their end point at 12 sessions [8], and outcomes at graduation for all other comparators, who used multiple end-point definitions [8,10,34,37].) When compared to naturalistic studies examining clinical outcomes of clinicians using a range of therapeutic modalities and approaches, response rates (46% in the literature vs 48% in this study) and recovery rates (21% in the literature vs 54% in this study) also compare favorably [11,37]. In anxiety disorders, a systematic review of outcomes for cognitive behavioral therapy showed reliable improvement rates of 45% (vs 62% in this study), and finally, a large naturalistic study showed recovery rates of 31% (vs 60% in this study) [10,38]. These results suggest that the outcomes observed in this study may be superior to outcomes observed in similar large-scale, real-world care settings and comparable to observed outcomes from RCTs of psychotherapy for depression and anxiety.

Rates of dropout and adherence to MBC in this sample also compare favorably to rates observed in the literature. The dropout rates in a standard health care setting were six times higher than observed in our sample (73.5% vs 10.1%) [6]. Completion rates of MBC surveys (PHQ-9 and GAD-7) were higher than 95% across the entire population, with individual clinicians having a median survey adherence rate of 90% within their patient panels. This is in stark contrast to general practice, where only 20% of providers use MBC at all, and 5% of practitioners use it in accordance with an empirically informed schedule (eg, every session) [17].

These findings suggest that in comparison to similar practice settings, patients in this study are more likely to receive an effective dose of care and achieve positive clinical outcomes. It is possible that the comprehensive use of MBC, supported by the suite of CDSS tools, enables clinicians to identify patients at risk of poor outcomes and adjust treatment in a timely manner, consistent with MBC best practices [17,18].

EBPs are known to be effective for improving overall outcomes in care; however, the financial, organizational, and clinician burden associated with the implementation and sustainment of EBPs likely contribute to their low uptake in clinical practice and limit their potential to improve outcomes [39]. The provision of clinically useful software that facilitates the practice of MBC may reduce some of the barriers to effective adoption and implementation. In this study, clinicians had access to consistent and automated measurement, visual interpretation guidance of results, and actionable insights, in addition to a set of other tools that improve accountability and may increase clinical efficiency and effectiveness. Although these tools cannot change clinical behavior on their own, they can support the practice of MBC and enable clinicians to provide more focused, evidence-based interventions. It is possible that these tools contributed to the high levels of patient retention and clinical outcomes observed in this study.

This study’s findings have implications for both value-based payment models as well as standard reimbursement models of care. Psychotherapy is an effective treatment for a broad range of mental health conditions, and health care claim–based analyses suggest that outpatient mental health treatment can save overall health care costs compared to those who do not receive treatment at all [40]. Importantly, evidence suggests that cost savings improve further if individuals are retained in care past one or two sessions, indicating that the dose of treatment must be sufficient to produce clinically meaningful change [40]. Moreover, emerging evidence suggests that decreases in symptoms drive reductions in costs associated with mental health conditions, particularly when compared to those whose symptoms stagnate or worsen over time [41]. Providing patients with the appropriate care, ensuring they remain engaged in care after initiation, and achieving meaningful outcomes can help reduce the overall cost and burden associated with mental health conditions.

### Limitations

Although this work offers some suggestive evidence that CDSSs may be associated with higher-quality practice, it is preliminary. The primary limitation of this study is the retrospective design and lack of a comparison group, which limits our ability to examine causal pathways. The study provides benchmarks for comparison to other general practice and structured psychotherapy outcomes; however, these are nonrandomized and unmatched comparators and thus are only illustrative of potential differences and should not be considered rigorous comparisons. Additional research using experimental designs and high-quality comparison samples is needed to isolate the specific impacts of the MBC and CDSS supports offered.

Second, there are numerous potential threats to generalizability outside of the study sample. The CDSSs were examined in one organization, and the study setting incentivizes clinician use of MBC, limiting generalizability to other organizations that may have differences in staffing, policy, culture, patient population, or other factors. The data may also only be generalizable to individuals with clinically elevated levels of depression and anxiety. In addition, to describe the outcomes of patients who received an adequate dose of treatment, the study excluded patients who dropped out prior to the fourth session, which may have inflated the results. Although lower than the results for treatment completers, most of these patients also experienced significant symptom reductions (55%), suggesting this limitation may be minor.

Third, the study design did not allow an examination of other factors that may have influenced the results, such as the clinician skill level, training effectiveness, or patient-level covariates (eg, baseline severity or concurrent treatment during the study period). Future studies should attempt to understand the contributions of these variables on clinical outcomes.

Finally, several authors are employed by the company where the data were generated, and although efforts were made to reduce potential bias by collaborating with external consultants, it is possible conflicts of interest may have influenced the results. A strength of the study is that the extremely high rate of measurement completion ensured that the outcomes of nearly all patients were observable, in contrast to other settings in which many patients may be lost to follow-up, providing a comprehensive view into outcomes in a naturalistic setting.

### Conclusion

The results of this retrospective cohort study suggest that an integrated software platform supporting MBC, clinical decision support, and automation of clinically burdensome tasks is associated with high levels of care engagement and clinical outcomes. Although additional research is needed to isolate and clarify the effects of these tools, these findings are suggestive that technology-enabled innovations may help improve the quality of care delivery at scale.

## Data Availability

The data presented are an accurate and honest account of the research performed and no data were fabricated or manipulated. Given the sensitive nature of the health care data used in the study, requests for raw data will be handled by the contacting author NRF and will be considered on a case-by-case basis.

## Appendix

Multimedia Appendix 1 Fixed effects output from longitudinal models of change in sessions 1-12 for the PHQ-9 (Patient Health Questionnaire-9) and GAD-7 (General Anxiety Disorder-7).

Multimedia Appendix 2 STROBE (Strengthening the Reporting of Observational Studies in Epidemiology) checklist.

## Abbreviations

CDSS: clinical decision support system
EBP: evidence based practice
HMO: health maintenance organization
GAD-7: General Anxiety Disorder-7
MBC: measurement-based care
MHQoL: mental health quality of life
PHQ-9: Patient Health Questionnaire-9
PPO: preferred provider organization
RCT: randomized controlled trial
