# Supplementary material for: Depression and Anxiety Outcomes in a Technology-Enabled Psychotherapy Practice: Retrospective Cohort Study
Source: JMIR Form Res. 2025 Dec 2;9:e76264. doi: 10.2196/76264 (PMC12709161; doi:10.2196/76264)
Supplement: Multimedia Appendix 1 [file formative_v9i1e76264_app1.docx]

**Multimedia Appendix 1.** Fixed effects output from longitudinal models of change in sessions 1-12 for the PHQ-9 (Patient Health Questionnaire-9) and GAD-7 (General Anxiety Disorder-7).

|  | **PHQ-9 model** | | | | **GAD-7 model** | | | |
| --- | --- | --- | --- | --- | --- | --- | --- | --- |
|  | **Estimate** | **Std. error** | **z value** | **Pr(>\|z\|)** | **Estimate** | **Std. error** | **z value** | **Pr(>\|z\|)** |
| **Intercept** | 14.80 | 0.15 | 100.07 | 0 | 14.29 | 0.14 | 105.34 | 0 |
| **Patient session number** | -1.00 | 0.02 | -41.64 | 0 | -1.06 | 0.02 | -43.87 | 0 |
| **Patient session number squared** | 0.05 | 0.00 | 26.33 | 0 | 0.05 | 0.00 | 28.26 | 0 |
| **Age 18 - 29** | 0.55 | 0.19 | 2.84 | 0 | 0.23 | 0.18 | 1.3 | 0.19 |
| **Age 50 - 69** | 0.39 | 0.22 | 1.79 | 0.07 | 0.30 | 0.21 | 1.44 | 0.15 |
| **Age 70+** | -0.34 | 0.43 | -0.79 | 0.43 | -0.48 | 0.41 | -1.17 | 0.24 |
| **Gender: man** | -0.06 | 0.19 | -0.29 | 0.77 | -0.35 | 0.18 | -1.94 | 0.05 |
| **Gender:**  **nonbinary/**  **trans/other** | -0.28 | 0.35 | -0.8 | 0.42 | -0.42 | 0.35 | -1.19 | 0.23 |
| **Gender: unknown** | -0.55 | 0.27 | -2.06 | 0.04 | -0.60 | 0.24 | -2.46 | 0.01 |
