# Supplementary material for: Depression and Anxiety Outcomes in a Technology-Enabled Psychotherapy Practice: Retrospective Cohort Study
Source: JMIR Form Res. 2025 Dec 2;9:e76264. doi: 10.2196/76264 (PMC12709161; doi:10.2196/76264)
Supplement: Multimedia Appendix 2 [file formative_v9i1e76264_app2.pdf]

STROBE Statement—checklist of items that should be included in reports of observational studies

	Item No.	Recommendation	Page No.
**Title and abstract**	1	*(a)* Indicate the study's design with a commonly used term in the title or the abstract<br>*Methods section of abstract includes direct statement about study design: "This retrospective cohort study"*	1
		(b) Provide in the abstract an informative and balanced summary of what was done and what was found<br>*We believe the abstract provides this level of information in alignment with journal guidelines*	1
**Introduction**			
Background/rationale	2	Explain the scientific background and rationale for the investigation being reported<br>*The introduction elaborates on the background and rationale with the importance of further research on MBC:"…access to effective, high quality mental health care remains problematic [5]." & "however, the implementation of these practices in mental health care can be hindered by cost, complexity, and challenges in sustaining the new clinical model [14,15]."*	2
Objectives	3	State specific objectives, including any prespecified hypotheses<br>*Following the scientific background, we specifically provide information on the current study goals and aims"The current study aims to…"*	6
**Methods**			
Study design	4	Present key elements of study design early in the paper<br>*The first sentence of the study design section is: "This is a retrospective cohort analysis focused on adult patients with clinically elevated symptoms of depression or anxiety who enrolled in mental health treatment with Two Chairs in the first half of 2024. "*	8
Setting	5	Describe the setting, locations, and relevant dates, including periods of recruitment, exposure, follow-up, and data collection<br>*Setting: "Patients were enrolled in behavioural health services at Two Chairs…"*<br>*Locations: "During the period of data collection, services were available in California, Washington, and Florida."*	8

		Relevant dates: "first half of 2024" & "first treatment session between January 1 and June 30, 2024"	
Participants	6	*(a)* *Cohort study*—Give the eligibility criteria, and the sources and methods of selection of participants. Describe methods of follow-up<br>*See Figure 1 and paragraphs 3 and 4 of the **Study Population** section*<br>*Case-control study*—Give the eligibility criteria, and the sources and methods of case ascertainment and control selection. Give the rationale for the choice of cases and controls<br>*Cross-sectional study*—Give the eligibility criteria, and the sources and methods of selection of participants	9, 11
		(b) *Cohort study*—For matched studies, give matching criteria and number of exposed and unexposed<br>*n/a*<br>*Case-control study*—For matched studies, give matching criteria and the number of controls per case	
Variables	7	Clearly define all outcomes, exposures, predictors, potential confounders, and effect modifiers. Give diagnostic criteria, if applicable<br>*This information is provided in the measurement section, including definitions of the patient demographics, screener tools utilized for measurement, and clinical outcomes*	13
Data sources/ measurement	8*	For each variable of interest, give sources of data and details of methods of assessment (measurement). Describe comparability of assessment methods if there is more than one group<br>*See the **Measurement** section where patient demographics are defined, screener tools are defined and explained, and clinical outcomes are described. Source of the data is also stated at the conclusion of the methods section.*	17
Bias	9	Describe any efforts to address potential sources of bias<br>*Controlling for patient demographics is described in the **Measurement** section. Further details about possible sources of bias and how they were mitigated against are presented in the ethics statement*	7-17
Study size	10	Explain how the study size was arrived at<br>*See Figure 1 and **Study Population** section*	9

Quantitative variables	11	Explain how quantitative variables were handled in the analyses. If applicable, describe which groupings were chosen and why<br>*Table 1 describes groupings*	18
Statistical methods	12	*(a)* Describe all statistical methods, including those used to control for confounding<br>*See statistical analysis section.*<br>*Confounders are controlled for: "…with random intercepts for clinicians and random intercepts and slopes (session number) for patients. Fixed effects included age, gender, session number, and a quadratic term for session number (Supplemental Table 1)."*	16
		(b) Describe any methods used to examine subgroups and interactions<br>Random effects are similar to interactions: "…*with random intercepts for clinicians and random intercepts and slopes (session number) for patients"*<br>*Subgroups: PHQ-9 and GAD-7 are described at the three time points (baseline, check-in session, and termination)*	17
		(c) Explain how missing data were addressed<br>*See Table 1*<br>*"Data is collected as a part of routine care and patients are not required to complete all questions. As a result, data sparseness varies across variables (see Table 1)." Additional details are also present in the methods section to provide narrative explanations about decisions on missing data.*	17
		(*d*) *Cohort study*—If applicable, explain how loss to follow-up was addressed. *In this study, loss to follow-up was not a topic of concern, as the decision was made to include either session 12 data or the last available assessment for the outcome variable, creating a dataset with no missing data due to loss of follow-up.*<br>*Case-control study*—If applicable, explain how matching of cases and controls was addressed<br>*Cross-sectional study*—If applicable, describe analytical methods taking account of sampling strategy<br>*n/a*	-
		(d) Describe any sensitivity analyses<br>*n/a*	-
Participants	13*	(a) Report numbers of individuals at each stage of study—eg numbers potentially eligible, examined for eligibility, confirmed eligible, included in the study, completing follow-up, and analysed	18

		"Among the 3,572 patients who met the initial criteria for inclusion, 89.9% (n=3,183) were retained to the 4th session and thus eligible for the primary analytic cohort. A small number of patients were excluded due to missing follow-up MBC scores or because their session 1 baseline was unavailable, leaving 2,984 patients (83.5% of the population meeting the original inclusion criteria, Figure 1). Patient demographics can be found in Table 1. Data for the clinical check-in session was most often collected from session 12 (71.4%) and the average check-in session number of 10.8. A total of 2,237 patients from the primary cohort terminated care during the observation window and therefore have termination outcomes. Of patients who completed their care, 38.1% did so by session 12 and 61.9% did so after session 12. The median termination session was 16 (average = 16.9). "		
		(b)	Give reasons for non-participation at each stage<br>"*A small number of patients were excluded due to missing follow-up MBC scores or because their session 1 baseline was unavailable*"	18
		(c)	Consider use of a flow diagram<br>*See Figure 1*	11
Descriptive data	14*	(a)	Give characteristics of study participants (eg demographic, clinical, social) and information on exposures and potential confounders<br>*See Table 1*	18
		(b)	Indicate number of participants with missing data for each variable of interest<br>*See Table 1*	18
		(c)	*Cohort study*—Summarise follow-up time (eg, average and total amount)<br>*Follow-up is defined as the clinical check-in session and these results are presented in Tables 2 and 3.*	21, 23
Outcome data	15*	*Cohort study*—Report numbers of outcome events or summary measures over time<br>*See Tables 2,3*		21, 23
		Case-control study—Report numbers in each exposure category, or summary measures of exposure		
		Cross-sectional study—Report numbers of outcome events or summary measures		

Main results	16	*(a)*	Give unadjusted estimates and, if applicable, confounder-adjusted estimates and their precision (eg, 95% confidence interval). Make clear which confounders were adjusted for and why they were included<br>*See Tables 2,3 and the paragraphs describing the tables to understand the confounders*	21, 23
		(b)	Report category boundaries when continuous variables were categorized<br>*Table 1 is very helpful to understand the boundaries*	18
		(c)	If relevant, consider translating estimates of relative risk into absolute risk for a meaningful time period<br>*n/a*	

Other analyses	17	Report other analyses done—eg analyses of subgroups and interactions, and sensitivity analyses<br>*All analyses are reported on and reported in both the primary text/tables/figures as well as in the supplemental materials*	
**Discussion**			
Key results	18	Summarise key results with reference to study objectives<br>*Restate study objectives: "The study was intended to…"*<br>*Summarize key results: "The findings indicate that…"*	26
Limitations	19	Discuss limitations of the study, taking into account sources of potential bias or imprecision. Discuss both direction and magnitude of any potential bias<br>*See **Limitations** section*	29
Interpretation	20	Give a cautious overall interpretation of results considering objectives, limitations, multiplicity of analyses, results from similar studies, and other relevant evidence<br>*Includes high level interpretation: "The results of this retrospective cohort study suggest that an integrated platform supporting measurement based care, clinical decision support, and automation of clinically burdensome tasks can produce improvements in care engagement and clinical outcomes."*<br>*Compares results to other literature, For example: "On average, improvement in PHQ-9 and GAD-7 scores exceeded the threshold for reliable and clinically significant symptom improvement on these measures.[35,36]…"*	26-29
Generalisability	21	Discuss the generalisability (external validity) of the study results<br>*"The CDSS systems were examined in one organization." Additional information about how the outcomes of this study may have limited generalizability.*	30
**Other information**			
Funding	22	Give the source of funding and the role of the funders for the present study and, if applicable, for the original study on which the present article is based<br>*See the Disclosure statement, which includes information about funding source.*	8

*Give information separately for cases and controls in case-control studies and, if applicable, for exposed and unexposed groups in cohort and cross-sectional studies.

Note: An Explanation and Elaboration article discusses each checklist item and gives methodological background and published examples of transparent reporting. The STROBE checklist is best used in conjunction with this article (freely available on the Web sites of PLoS Medicine at http://www.plosmedicine.org/, Annals of Internal Medicine at http://www.annals.org/, and Epidemiology at http://www.epidem.com/). Information on the STROBE Initiative is available at www.strobe-statement.org.
